# Conscientious objection to abortion: why it should be a specified legal right for doctors in South Korea

**DOI:** 10.1186/s12910-020-00512-3

**Published:** 2020-08-06

**Authors:** Claire Junga Kim

**Affiliations:** Department of Medical Education, Ewha Womans University College of Medicine, 52 Ewhayeodae-gil, Seodaemun-gu, Seoul, 03760 Republic of Korea

**Keywords:** Conscientious objections, Abortion, Decriminalization of abortion, Moral integrity, No-impediment condition

## Abstract

**Background:**

In 2019, the Constitutional Court of South Korea ruled that the anti-abortion provisions in the Criminal Act, which criminalize abortion, do not conform to the Constitution. This decision will lead to a total reversal of doctors’ legal duty from the obligation to refuse abortion services to their requirement to provide them, given the Medical Service Act that states that a doctor may not refuse a request for treatment or assistance in childbirth. I argue, confined to abortion services in Korea that will take place in the near future, that doctors should be granted the legal right to exercise conscientious objection to abortion.

**Main text:**

Considering that doctors in Korea have been ethically and legally obligated to refrain from abortions for many years, imposing a universal legal duty to provide abortions that does not allow exception may endanger the moral integrity of individual doctors who chose a career when abortion was illegal. The universal imposition of such a duty may result in repudiation of doctors as moral agents and damage trust in doctors that forms the basis of medical professionalism. Even if conscientious objection to abortion is granted as a legal right, most patients would experience no impediment to receiving abortion services because the healthcare environment of Korea provides options in which patients can choose their doctors based on prior information, there are many doctors who would be willing to provide an abortion, and Korea is a relatively small country. Finally, the responsibility to effectively balance and guarantee the respective rights of the two agents involved in abortion, the doctor and the patient, should be imposed on the government rather than individual doctors. This assertion is based on the government’s past behaviours, the nature of its relationship with doctors, and the capacity it has to satisfy both doctors’ right to conscientious objection and patients’ right to legal medical services.

**Conclusion:**

With regard to abortion services that will be sought in the near future, doctors should be granted the legal right to exercise conscientious objection based on the importance of doctor’s moral integrity, lack of impediment to patients, and government responsibility.

## Background

In 2019, the Constitutional Court of South Korea held that the country’s anti-abortion provisions do not conform to the Constitution [1]. In Korea, having an abortion has been treated as a criminal offense [2], except for a few special cases [3]. Article 269 Paragraph 1 of the Criminal Act, namely the Self-Abortion Provision, penalizes a pregnant woman who procures her own abortion, and under Article 270 Paragraph 1 of the Criminal Act known as the Abortion by Doctor Provision, doctors can be punished for performing an abortion on a woman upon her request or consent [2]. A trial was held to review the constitutionality of these two provisions, resulting in a ruling that they did not conform to the Constitution [1]. This type of court decision allows the law to remain in effect until revised to avoid social confusion and the legal vacuum that can result from immediate invalidation of the law. The Constitutional Court ordered abolition of the anti-abortion provisions and their amendment by December 31,2020 [1].

This court decision is a complete reversal of the stance that South Korea’s legal system has maintained thus far. Since its inception in 1953, the Criminal Act of Korea has criminalized abortion. Although the Mother and Child Health Act, which was legislated in 1973 as part of the anti-natalist policy designed to motivate economic development [4], allows a woman to terminate her pregnancy within 24 weeks of conception, it is only under following circumstances prescribed by the Act that abortion may be carried out: where her or her spouse suffers from any eugenic or genetic mental disability or physical disease prescribed by Presidential Decree; where her or her spouse suffers from any contagious disease prescribed by Presidential Decree; pregnancy from rape or quasi-rape; pregnancy from sexual relations between blood relatives or matrimonial relatives who are legally unable to marry; and where the maintenance of the pregnancy damages or is likely to damage the health of the mother due to health or medical issues [3]. However, the act does not consider social and economic determinants of abortion or severe foetal medical conditions as permissible grounds for abortion, which has rendered the vast majority of abortions illegal. Furthermore, in 2012, the Constitutional Court upheld the anti-abortion provisions, acknowledging the right to life of a foetus and declaring that the right to life of a foetus takes precedence over a pregnant woman’s freedom to choose abortion based on her right to self-determination [5]. Also, the court did not provide for differential protection of a foetus according to stage of growth, making it illegal to abort even an early-stage pregnancy [5, 6].

However, in 2019, the Constitutional Court concluded that the anti-abortion provisions in the Criminal Act, both the Self-Abortion Provision and Abortion by Doctor Provision, violate the principle of proportionality as they overly infringe on a pregnant woman’s right to self-determination. The court also decided that the value of life can vary depending on stage of foetal development; and that in consideration of this, there must be a balance between a foetus’ right to life and a woman’s right to self-determination. In addition, 22 weeks of gestation, at which stage a foetus is considered viable or able to survive on its own, was proposed as the point of pregnancy before which abortion is permitted. The court decided that abortion for social and economic reasons should be permitted as well [1]. Therefore, the Criminal Act will be revised based on this decision, decriminalizing abortions which were previously treated as illegal.

With abortion decriminalized, pregnant women will be able to undergo an abortion and doctors will be placed in the position where they must perform an abortion. Thus far, doctors have not been required to carry out an abortion except under the particular circumstances enumerated in the Mother and Child Health Act, as they had legal obligations to equally protect the life of a pregnant woman and the life of a foetus. However, when abortion becomes a legal medical service based on the 2019 decision by the Constitutional Court and subsequent amendment of the Criminal Act, doctors will not only have a professional obligation, but also a legal duty to provide an abortion. Under Article 15 ‘Prohibition against Refusal to Provide Medical Examination or Treatment’ of the Medical Service Act [7], doctors are not allowed to reject a request for medical treatment, or assistance in childbirth without justifiable grounds. Here, “medical treatment, or assistance in childbirth” is a legal phrase that encompasses all medical services which can be rendered based on the capacity of the requested doctor, and this would also come to include the abortion service once legalized. Moreover, according to the authoritative interpretation [8], “justifiable grounds” for rejection of medical service correspond to circumstances where rendering of medical service can do harm to the patient due to the absence of adequate facilities, physical illness of the requested doctor, and so on. However, objection on the grounds of conscience is not recognized as a ground for justification. Violation of Article 15 is punishable by imprisonment or a fine. Therefore, based on the Medical Service Act, a doctor’s legal duty will be reversed from a duty not to perform an abortion to a duty to perform an abortion.

In South Korea, the concept of conscientious objection is almost non-existent in healthcare, or at least there has been no serious discussion of it. Conscientious objection has a long history in the West, dating back to the time of colonial militias, and has almost reached the status of a recognized right for individuals [9]. In Western societies, a doctors’ right to conscientious objection is often protected by law as well [9, 10]. However, in Korea, conscientious objection is barely recognized. To be clear, Article 6 of the Framework Act on Health and Medical Services stipulates that healthcare and medical practitioners have the right and duty to provide health and medical services based on their knowledge, experience, and conscience [11]. However, it does not specify to what extent doctors are allowed discretion in acting on their conscience nor is there any judicial precedent to clarify the matter [12]. Also, there has never been a case where conscientious objection regarding abortion or life-sustaining treatment by medical professionals was granted by law or professional guidelines.

Given that the concept of “conscience” lacks sufficient detail in the law, although mentioned in the Framework Act on Health and Medical Services, and that doctor’s rejection of medical services in violation of Article 15 of the Medical Service Act can lead to a punishment of imprisonment or a fine, it would be rational for the doctors to believe that their right to conscientious objection is not being effectively guaranteed. A widespread perception in Korea is that, when a certain act is called for legally and ethically, it should be equally applied to every relevant person. The notion that when the belief of an individual conflicts with that of the majority, the former should also be respected as much as possible is quite novel to many people in Korea. This is evidenced by the fact that the Constitutional Court and the Supreme Court only recently ruled in favour of conscientious objection to military service [13, 14]. Thus, it is fair to say that, while the West is mainly concerned about conscience creep [15] or conscience without consequence [16], Korea is just beginning to acknowledge individual rights.

In this paper, I will not discuss the ethical status of abortion or conscientious objection in general. Of course, how much importance would we have to grant to the matter of doctors’ conscience and one’s moral judgement is indeed a bigger question that deserves our attention when examining the issues that this paper seeks to address. And there has been much debate across different countries over the topic of conscientious objection in many different areas not only against abortion [17–26] or, on the contrary, ban on abortion [27], but also against emergency contraception [17, 28, 29], oral contraceptives [17, 23], and physician assisted suicide [23–26, 30]. However, as the goal of this paper lies in making a moral argument about conscientious objection building on from the assessment of the specific circumstances in Korea, covering all the relevant debates aforementioned was impossible due to the limits of space. In particular, an obligation that comprehensively bans doctors from rejecting patient’s request for medical treatment exists in Korea, requiring that doctors render all medical treatments requested by patients, whether emergency patient or not, unless there are justifiable clinical grounds. The obligation imposed on the doctors is accompanied by a penalty clause, which makes Korea’s situation very different to that of other countries. Hence, this paper concentrates on the analysis of unique situation in Korea, arguing for the legal right to conscientious objection to abortion. Still, it is my hope that this paper which argue for the legal right to conscientious objection to abortion in Korea, would be relevant in covering many, if not all, major issues that arise in the discussion of conscientious objection.

I will confine the scope of the argument to legal regulations on abortions services that will be sought in the near future in South Korea. However, the right to reject abortion services requested by the patient on clinical grounds—the right that physician decline the requested abortion or the requested certain type of abortion method which is clinically counter indicated due to the expected side effects [31–35] and unfavourable risk and benefit ratio in each case—will not be covered in this paper. Refusing treatment requested from patients on clinical grounds is recognized as one of the justifiable grounds that exempt the physicians from legal punishment with regards to the Article 15 of Medical Service Act, ‘Prohibition against Refusal to Provide Medical Examination or Treatment [36].’ Therefore, this issue lacks urgency compared to the treatment refusal on conscientious grounds which is hardly recognized or discussed in both the academia and field of practice in Korea. Thus, narrowing the scope of discussion, I argue that a doctor in Korea should be given the legal right to refuse to provide abortion services based on his or her conscience. There are three reasons for this. First, forcing all involved medical professionals, specifically obstetricians who are capable of and have the resources to provide abortion services, to offer abortion services poses a moral and professional threat not only to individual practitioners who oppose abortion, but also to the entire medical profession. Second, recognizing the legal right to conscientious objection to abortion will not seriously infringe on patient rights to receive the desired treatment. Third, it is the responsibility of the government to guarantee protection of doctor and patient rights, not that of individual doctors.

## Main text

### Moral integrity

The importance of moral integrity for medical professionals, both individual medical professionals and the medical profession in a collective sense, justifies the granting of legal rights of conscientious objection to abortion to medical professionals in Korea. With regard to acts that are closely related to one’s core ethical values such as abortion, exercise of conscience is indispensable for doctors to protect and maintain their moral integrity [37]. If a certain issue is grave enough to be linked to core ethical values, or to a deeply held conviction [38], then it is a matter of conscience. For instance, when life or death is at stake [26], most people would consider the matter to be tied to their core values. In this regard, it is reasonable to say that, when confronted with the choice of whether to perform an action, for example, whether to participate in execution of the death penalty, many people are inclined to heed the voice of their conscience. No one would object to the statement that abortion is related to core ethical values, although there is a huge gap in views between those in favour and those against abortion. This is because abortion concerns both the life of a foetus potentially capable of becoming a person and the right to self-determination, which is one of the most fundamental ways for an individual to exercise one’s personhood. The belief system of an individual can be likened to a house built on these core ethical values as pillars. If these values are broken, an individual’s belief system may collapse.

If, when faced with a problem related to core ethical values, an individual is prevented from acting in accordance with his or her beliefs of what is right, that person is bound to feel ‘guilt and shame, a sense of self-betrayal and personal disintegration, and loss of self-respect [39]’. If this negative experience occurs repeatedly, he or she could end up feeling like an insignificant part of a larger, irreformable structure or accepting the experience as an inevitable result of natural events, rather than viewing himself or herself as a moral agent.

The logic of associating an act that goes against one’s conscience with the repudiation of agency implied in an actor’s personhood also applies in Korea.. The Constitutional Court defines the conscience it seeks to protect as a ‘strong and serious voice of mind that says in judging the right and wrong of a certain act, doing otherwise can result in a collapse of one’s existential value as a person [40]’. Conscience, as seen from the Constitutional Court’s definition, is extremely valuable as it is what allows an individual to recognize oneself as a moral agent. Therefore, it is of great benefit to allow doctors who believe that performing an abortion could disrupt the core ethical pillars upholding their belief system to exercise conscientious objection.

Among acts of conscience, conscientious *objection* arises when an act at issue arouses controversy, that is to say, when an act is of uncertain moral status [38] or triggers ‘real debate’ [24] for and against it among members of society. Acts that everyone without doubt agrees as moral are not subject to conscientious objection. Refusing such an act is immoral and cannot be seen as being in accordance with conscience. Abortion, however, has been a subject of controversy for many years, and this controversy is unlikely to die down, even with new embryological findings. It is conceivable that two different doctors who both seek to promote the good of a patient may have opposite ideas on their obligation in relation to abortion. In other words, while one doctor may think that it is his or her moral duty to perform an abortion procedure even when illegal [27], the other may refuse to provide an abortion service even if it may result in imprisonment, based on the belief that protecting a potential human being is a moral duty. In this regard, a doctor’s conscientious objection to abortion should be respected.

When discussing whether to grant doctors the right to exercise conscientious objection to abortion, it is important to consider that the doctors did not expect the specialty or work environment they chose would involve abortion. Some have argued that medical professionals need to accept the duties implied in their voluntary choices because they chose their profession or specialty freely [25, 30, 41]. Still, is it right to view the decision to become a doctor as blanket acceptance of the time-transcending obligation to provide all medical services that are recognized as legal at any point in time? What if duties regarding a certain task at the time when a profession or specialty was chosen and the newly imposed duties for the same task are exactly opposite?

As an example, consider a person currently practicing as an obstetrician. Reflect on the time when she was a medical student who wished to become an obstetrician out of a desire to help life come into the world. This student is concerned about the possibility of having to perform an abortion, which is against her core ethical values. Unsurprisingly, for this medical student, the illegal status of abortion at the time when she made the decision to become an obstetrician was of great importance. After all, those past circumstances led her to expect that she would be able to rightfully refuse a request by a patient for an illegal abortion service. It is with this expectation that she chose the path to become an obstetrician. Those doctors who made the decision to become an obstetrician during the time when abortion was a criminal offense should not be obligated to provide abortion services that were legalized later, at least not for the reason that it was their choice to become an obstetrician. Today, doctors in Korea are faced with the abruptly imposed obligation to provide abortion services. It is nonsensical to say to these doctors, ‘The moment you chose to be an obstetrician, you also chose to accept the duty to provide abortion services, which were illegal at the point of your decision but have since been legalized.’

Dramatic changes in norms are expected to take place not only in the legal sphere, but also in the medical profession. Given the confusion these rapid changes will bring about, conscientious objection can have a positive impact on medical professionalism. Many studies have focused on the negative impacts of conscientious objection on professional obligation [21, 23, 25, 30]. It is true that conscientious objection presents some danger to the fulfilment of professional obligation to provide the treatment that patients deserve. When it comes to abortion in Korea, however, because of the anti-abortion norms in the profession that have been maintained to date, allowing a few to exercise conscientious objection to providing abortion services can be of some help to medical professionalism. Paragraph 1 of Article 33 ‘Ethics Related to the Foetus’ of the Korean Medical Association’s Code of Medical Ethics states that ‘doctors should do their best to preserve the life and promote the health of a foetus,’ and the second paragraph of the same article stipulates that ‘doctors should use prudence in performing an artificial termination of pregnancy even when it is medically appropriate and reasonable, and should pay special attention to the health of the mother and the right to life of the foetus [42].’ Considering this Article alone, professional norms do not even treat a pregnant woman’s right to self-determination in abortion as ethically relevant. Furthermore, as indicated by the obligation to be prudent with respect to carrying out an abortion *even under a medically appropriate situation*, an anti-abortion stance with emphasis on the foetus’ right to life is prevalent. In fact, in the previous version of the Code of Medical Ethics, such a stance was even more evident. Paragraph 2 of Article 15 ‘Protection of Foetus and Prohibition of Sex Discernment’ stated that ‘doctors shall not artificially terminate pregnancy for purposes other than those legally and medically recognized for protection of the life, health, and personhood of a foetus and a pregnant mother [43].’ Such Article shows that the medical professionalism demanded in the professional norm used to be a mere parroting of the demands made in the anti-abortion provisions in Criminal Act, without a further consideration or modification. In short, South Korea’s professional medical norms to date have placed great emphasis on the professional duty not to carry out an abortion.

Wicclair says that conscientious objection deserves more far-reaching recognition if it heads in the same direction as professional norms [37]. When many people judge a certain act or refrainment from an act to be in alignment with the core values of medicine, and such judgment is shared by the large majority of the members of the profession, the act or refrainment from the act may be elevated to a professional norm. When such elevation takes place, anyone who seriously intends to practice medical professionalism becomes more strongly motivated to carry out or refrain from the act. Moreover, one can often go so far as to integrate what is demanded by the professional norms with one’s personal belief system, and finally internalize these norms. In other words, some medical professionals in Korea are likely to have already developed a conscience that equates objection to abortion as the duty of medical practitioners because of the medical training they received and their sincere aspirations for medical professionalism. Telling such a professional to ‘just do your job [44]’ can be considered an assault on him or her as an individual. Moreover, it can be considered a sign of disrespect for all medical professionals as they are not granted even minimum autonomy as professionals.

There is a fundamental reason why conscientious objection deserves support in terms of medical professionalism. Existence of a system that regards doctors’ moral integrity so highly that it grants doctors even the right to exercise conscientious objection is evidence that doctors are considered moral agents. In fact, moral agency is a precondition for every adult-to-adult relationship in its true sense. Only in a relationship between moral agents can people truly have expectations of the other person and give praise or blame [45]. That is to say, awareness that a certain person is accountable for his or her act as a moral agent makes it possible for one to trust the moral agent and enter into a relationship. In this regard, moral integrity benefits not only the possessor, but also the people with whom the possessor interacts [38].

As medicine is a moral enterprise and doctors have fiduciary duty, acknowledging doctors as a moral agent is all the more important [46]. If doctors consider themselves mere tools for policy or law, argue that they are neither willing nor able to make a judgment regarding the moral quality of the medical practice they perform, and eventually claim that they are not responsible for the medical practice they provided or did not provide and its consequences, is it possible to trust those doctors? Unless we fully rely on robot-doctors for every decision and action related to healing, the answer is no. There is no evidence that doctors who practice conscientious objection are more ethical than those who do not. However, when there is an abrupt change in legal obligations, a medical system that allows conscientious objection serves to reaffirm the role of doctors as professionals accountable for their medical practice, not ones who blindly follow laws and policies. This reaffirmation would strengthen trust, the cornerstone of the doctor-patient relationship.

Although it can be argued that a system that grants conscientious objection can have a positive impact on medical professionalism, conscientious objection poses an unavoidable threat to professional obligation to provide treatment for patients. In this light, I do not argue that moral integrity should always take precedence over professional obligation to provide treatment. Undeniably, medical professionals enjoyed privileges during the course of acquiring the knowledge to become a doctor, and such privileges generate a professional obligation to provide medical services to patients who need them [46]. In this regard, it must be determined whether conscientious objection can be exercised in a manner compatible with professional obligation. If it can, then it can be justified; the positive impact of conscientious objection on professionalism discussed above provides additional justification. I will demonstrate in the following section that, considering the medical environment and the government’s role, conscientious objection to abortion can be exercised in a manner compatible with professional obligation in Korea.

### No impediment condition

Conscientious objection inevitably causes some degree of harm to patients for the sake of doctor’s liberty. Therefore, the amount of harm caused to patients needs to be measured before deciding to guarantee conscientious objection as a legal right. I accept Wicclair’s compatibility thesis that, in a certain environment, conscientious objection is compatible with doctors’ professional obligations [47, 48]. On the contrary, if a patient needs and deserves a certain medical service but is hindered from receiving it due to conscientious objection, then such objection is incompatible with fulfilment of professional obligation, and justification of conscientious objection weakens as much.

My aim in this section therefore, is not to determine whether conscientious objection is right or wrong per se, but whether conscientious objection, *substantively,* constitutes no impediment [49, 50] to patients who wish to receive abortion services in Korea’s medical practice environment. In this regard, it is important to assess whether there is room in Korea’s medical environment for medical professionals to fulfil their collective duty to provide treatment to patients while allowing some individual doctors to act according to their conscience. My answer to this question is yes, and the reasons are as follows.

First, patients in Korea have relatively great freedom to choose their doctors. There is no general physician system in Korea, and all medical institutions are accessible within the National Health Insurance system [51]. Furthermore, government organizations report specific information about the various medical institutions on their websites, such as antibiotic administration rate [52]. This ensures that, even if conscientious objection is recognized as a legal right, a requirement to inform the government and patients of the intent to exercise this right will effectively guarantee patients adequate options. For instance, the government can gather information about the current status of medical institutions with regard to applications for conscientious objection to abortion and disclose this on their website or by mail. This will allow patients to find doctors who provide abortion services. Such prior information can be made available not only on government organizations’ websites, but also on hospital websites, at hospital entrances, and so on. With these real options, there will be no impediment to accessing the abortion services that they need, want, and deserve. Patients will be able to effectively determine those doctors who do not provide treatment at the earliest possible stage before they enter into a doctor-patient relationship. Through this sorting process, they can avoid the feelings of rejection or moral criticism that may arise when they are refused treatment or are referred.

Second, there are unlikely to be many potential applicants who are willing to exercise conscientious objection to abortion, which buttresses the argument that adequate options will be available for patients. In Korea, debate over abortion has developed differently from that in the West. Attempts to inflict actual harm to the other side as well as the hatred that has infected the debate in the West are almost non-existent in Korea. In one empirical research study, Korean Evangelical Protestants and Roman Catholics reported more liberal views on abortion than those in the USA or Philippines [53]. One can assume that the extent of polarization on this issue is much smaller in Korea. Of course, this is not to say that there has been no moral criticism of women who have had abortions. Obviously, the anti-abortion provisions and negative moral judgment of abortion have been used as mechanisms not only to condemn, but also to threaten to punish these individuals. Abortion, however, is considered a women’s issue. Ironically, there has been little moral condemnation of doctors who perform abortions. Abortion has not been politicized until recently and has remained outside the public’s attention [54]. Furthermore, according to a recent study conducted by a government research institution, 21% of the respondent women reported having experienced an abortion [55]. As demonstrated by this study, many doctors perform abortion procedures despite their illegal status, which makes it possible to anticipate that most doctors will provide abortion services when abortion becomes decriminalized.

Third, the geographic environment of South Korea is such that the vast majority of patients will be able to access abortion services. Korea is a relatively small country, with a territory of 100,378km^2^, and most of the population, especially women of childbearing age, live in urban areas where they have access to medical services. Lack of access to medical services due to geographical limitations is unlikely to be an issue in Korea, unlike in the Northern part of Canada [19]. Granted, there is regional disparity in access to medical services in Korea. In particular, immigrant women, a substantial proportion of whom reside in rural areas, are likely to experience difficulty accessing care due to two simultaneous impediments; relatively low geographic access to medical services is one, and the other is that they may lack understanding about what kind of medical services they can receive due to their unfamiliarity with the language and culture. This problem and how to address it will be discussed in ‘Government’s responsibilities,’ in more detail. Generally, however, it is fair to say that Korea’s relatively small land size and urbanized infrastructure will help ensure that conscientious objection does not result in significant infringement of a patient’s right to abortion services.

### Government’s responsibilities

With regard to abortion, the Korean government is responsible for protecting both doctors’ legal right to exercise conscientious objection and patients’ right to medical services that are guaranteed under the law. The issue of abortion is not something that can be resolved by placing the burden solely on the shoulders of the doctors by requiring uniform treatment with no discretionary power. The government is responsible for making sure that the freedom of doctors and that of patients are in balance and do not excessively infringe on each other. The government must review the actual accessibility of medical services if it is to grant conscientious objection, which is likely to provide an even better guarantee of patients’ right to receive the treatment they deserve. All of these are government responsibilities. There are three reasons that the government should take on these responsibilities: the government’s past indecisiveness, the nature of its relationship with doctors, and the capacity it has for satisfying both doctors’ right to exercise conscientious objection and patients’ right to receive legal medical services.

Until very recently, the government has used anti-abortion provisions at its convenience. Anti-abortion provisions have remained in place since they were adopted in 1953. However, the Korean government has arbitrarily ignored or used these provisions for population control. In the 1960s and 1970s, during the early industrialization period when the government sought to reduce population size, it turned a blind eye to abortion or even encouraged it [56]. It was under this very context that the Article 14 ‘Limited Permission for Induced Abortion Operations’ of the Mother and Child Health Act was legislated [4]. A very small number of people were indicted for abortion, indicating that it was in effect a dead letter [54, 57]. As Korea’s birth rate has recently fallen to the world’s lowest [58], the government declared to designate abortion surgery as an ‘act of immoral treatment’ in the Medical Service Act, punishable by license suspension, and cautioned that a doctor who performs abortion surgery will face punishment [59]. This sudden policy change was an attempt to increase the birth rate. The government’s arbitrary use of anti-abortion provisions to control women’s bodies in a way that best suits their policy objectives is partially responsible for the legal confusion surrounding abortion.

Given the inconsistency of the government in dealing with anti-abortion provisions, it is unfair to delegate the burden of providing abortion services only to doctors. Legal recognition of abortion services as a treatment is a dramatic change, and it must be determined who will bear the burden caused by this change. It was the Korean government that introduced this change through the court’s decision. Additionally, the Korean government has caused confusion among citizens for many years by maintaining but arbitrarily enforcing the anti-abortion provisions. In short, because of past actions of the Korean government, it is responsible for the burden caused by abrupt changes surrounding anti-abortion provisions.

Considering the contribution of the government to fostering doctors and setting up medical facilities, the government cannot force doctors to provide uniform treatment against their conscience. Savulescu and Schuklenk argue that doctors cannot claim conscientious objection given the privileges they have received [23]. Here, I will not make a judgment on whether doctors in public medicine such as those in the United Kingdom (UK) should be given the right to conscientious objection. However, what should be noted is that the relationship between the government and the doctors in Korea, which is quite different from that in the UK, cannot justify imposition of a strong obligation by the former on the latter. Korea does not have a public medicine system as systematic as that in the UK. It is not the government but individual doctors and would-be doctors who bear the bulk of the costs incurred to receive training for becoming a doctor and setting up medical facilities. If the obligation of doctors to provide uniform treatment varies in proportion to social privilege, doctors in Korea should be given more freedom than those in countries such as the UK where public medicine plays a large role.

The Korean government has the capacity to satisfy both doctors’ right to conscientious objection and patients’ right to legal medical services. Furthermore, only the government has such capacity, not the doctors, either individually or collectively. Earlier in the ‘No impediment condition,’ I argued that granting the right to conscientious objection will not significantly impede women’s access to abortion services because Korea is relatively small and mostly urbanized. Still, even in this small country, there are areas and populations with low medical access due to geographic and cultural factors. The only entity that can address this issue is the government, which will have to utilize its public beds more actively to provide abortion services. There are a total of 221 public hospitals in South Korea, most of which are in major provincial cities [60]. These hospitals should provide abortion services and also relevant guidance about services for local residents. Policies should be designed to reflect the needs of the vulnerable who experience difficulties finding the treatments they deserve, such as immigrant women in rural areas. Furthermore, by specifying the obligation to provide abortion services as a contract term, the government can find doctors who will voluntarily perform the procedure in public hospitals. This is a better way to guarantee abortion services than forcing all doctors to provide these services, and a better way than idly letting them make excuses and not provide the services.

By stipulating the legal right to conscientious objection and accompanying obligations, the government can not only establish a balance between doctors’ and patients’ rights, but also protect both doctors and patients effectively. The government can grant conscientious objection to abortion to doctors as a legal right and specify accompanying obligations. To make it possible for doctors to protect their moral integrity and for the no impediment condition to be satisfied, the following conditional obligations should be put in place. If a doctor seeks to exercise conscientious objection to abortion, he or she should report his or her intention to do so to the government in advance. At this time, the reason for objection should be declared and deemed valid; it should not be because one wants to avoid time and effort involved in providing less profitable procedures or simply feeling reluctant, but because the act of providing an abortion conflicts with one’s core ethical values within a belief system, which is to say that it is a matter of conscience. Also, he or she should agree to reveal his or her intention to exercise conscientious objection to potential patients, possibly before the treatment relationship begins, and for the government to make public whether he or she is a conscientious objector. In addition, he or she should inform a patient in need of abortion services of their legality and refer the patient to a doctor who can help. Doctors may have the right to conscientious objection but having that right does not mean that the right of the patients to receive treatments that they deserve can be dismissed. On the contrary, the reason why doctor’s right to conscientious objection is recognized and can practically be exercised is because of the no impediment condition, meaning that it is possible for patients to receive the treatments which they deserve from other doctors within the medical system. Law, code of ethics, positional statement, or guidance of professional body in other countries require doctors to ensure the continuity of patient treatment even when their right to conscientious objection is exercised [61–66]. If conscientious objection against abortion is to be recognized as a legal right in Korea, then the doctor’s duty to inform a patient, and to refer the patient to another doctor should be stated explicitly to balance the legal rights of doctors and patients. In an emergency situation, however, where a pregnant woman’s health or life is in danger, the duty to save the patient’s life should take precedence over the right to conscientious objection. By providing the legal right to conscientious objection along with a list of obligations that a doctor should follow if she or he is to exercise this right and define its specific scope, the government can maintain the no impediment condition effectively. Not only that, if doctors file prior reports of their intention to exercise conscientious objection, the government can review the actual accessibility of abortion services and complement deficiencies where needed. After identifying areas with an inadequate number of doctors that provide abortion services, the government should ensure that doctors willing to perform abortions are hired in public hospitals and public clinics in that area. As demonstrated, providing and managing the legally stipulated right to conscientious objection is a much more effective way to guarantee patients’ right to medical services than forcing doctors to provide these services.

## Conclusions

Policymakers in South Korea should guarantee the legal protection of doctors to conscientious objection by including it in Article 15 ‘Prohibition against Refusal to Provide Medical Examination or Treatment’ of the Medical Service Act in the form of an exception. Considering that doctors have been ethically and legally required to refrain from abortions since 1953 in Korea, obligating every doctor to provide abortions jeopardizes the moral integrity of individual doctors who made the career choice at the time when abortion was illegal. It also can result in these doctors not being recognized as moral agents and damaging trust, which underlies medical professionalism. Even if conscientious objection to abortion is legally recognized, most patients would experience no impediments to abortion services because of the healthcare environment in Korea. There is an adequate number of accessible doctors they can choose by referring to prior information, there will be a large number of doctors who would provide abortions, and Korea is a relatively small country with an urbanized infrastructure. Lastly, to hold the government accountable for its past indecisiveness on abortion and to treat both doctors and patients fairly, policymakers should allow the legal right to conscientious objection. The right to conscientious objection with accompanying obligations is a tool that can both achieve balance between the two agents involved in abortion and guarantee their rights effectively.

## Data Availability

Not applicable.
